# Next Generation Sequencing of Chromosome-Specific Libraries Sheds Light on Genome Evolution in Paleotetraploid Sterlet (*Acipenser ruthenus*)

**DOI:** 10.3390/genes8110318

**Published:** 2017-11-10

**Authors:** Daria A. Andreyushkova, Alexey I. Makunin, Violetta R. Beklemisheva, Svetlana A. Romanenko, Anna S. Druzhkova, Larisa S. Biltueva, Natalya A. Serdyukova, Alexander S. Graphodatsky, Vladimir A. Trifonov

**Affiliations:** 1 Institute of Molecular and Cellular Biology Siberian Branch of the Russian Academy of Sciences, 630090 Novosibirsk, Russia; alex@mcb.nsc.ru (A.I.M.); bekl@mcb.nsc.ru (V.R.B.); rosa@mcb.nsc.ru (S.A.R.); rada@mcb.nsc.ru (A.S.D.); bilar@mcb.nsc.ru (L.S.B.); ns3032@yandex.ru (N.A.S.); graf@mcb.nsc.ru (A.S.G.); vlad@mcb.nsc.ru (V.A.T.); 2 Synthetic Biological Unit, Novosibirsk State University, 630090 Novosibirsk, Russia

**Keywords:** sturgeon, spotted gar, paralog chromosomes, whole genome duplication, synteny, fluorescence in situ hybridization, high-throughput sequencing

## Abstract

Several whole genome duplication (WGD) events followed by rediploidization took place in the evolutionary history of vertebrates. Acipenserids represent a convenient model group for investigation of the consequences of WGD as their representatives underwent additional WGD events in different lineages resulting in ploidy level variation between species, and these processes are still ongoing. Earlier, we obtained a set of sterlet (*Acipenser ruthenus*) chromosome-specific libraries by microdissection and revealed that they painted two or four pairs of whole sterlet chromosomes, as well as additional chromosomal regions, depending on rediploidization status and chromosomal rearrangements after genome duplication. In this study, we employed next generation sequencing to estimate the content of libraries derived from different paralogous chromosomes of sterlet. For this purpose, we aligned the obtained reads to the spotted gar (*Lepisosteus oculatus*) reference genome to reveal syntenic regions between these two species having diverged 360 Mya. We also showed that the approach is effective for synteny prediction at various evolutionary distances and allows one to clearly distinguish paralogous chromosomes in polyploid genomes. We postulated that after the acipenserid-specific WGD sterlet karyotype underwent multiple interchromosomal rearrangements, but different chromosomes were involved in this process unequally.

## 1. Introduction

Whole genome duplications (WGDs), the events resulting in polyploid organisms’ appearance, are not very common among chordate animals (in contrast to plants, for instance) [1], but they played a substantial role in early vertebrate evolution [2,3]. There were two rounds of ancestral WGD in the common ancestor of vertebrates (1R and 2R), which occurred about 500–600 million years ago (Mya) [4,5,6]. Teleosts (bony fishes) underwent their own lineage-specific WGD (TS3R, teleost-specific 3R) about 320 Mya [4]. Despite possible problems with epigenetic regulation and meiosis, polyploidizations increase genome plasticity, thus facilitating diversification [1]; therefore, polyploids might become more successful than their non-polyploid relative species in specific circumstances, like mass extinctions and the arising of new ecological niches [3].

Polyploidy among extant vertebrate animals quite often occurs in ray-finned fishes and amphibians [3], but it is lacking in mammals and birds (probably because of their sensitivity to gene dosage and other epigenetic effects [1,3]). WGDs and subsequent rediploidization (chromosomal rearrangements together with gene neofunctionalization, subfunctionalization or loss) are the phenomena of interest in evolutionary studies. There are several polyploid animal species with sequenced and assembled genomes [7,8], although the genomic analysis is very challenging in such cases [9]. Paralogous and homeologous chromosomal regions can be revealed by methods based on the comparison of certain genes numbers, conserved synteny, expression levels or pseudogenization [7,8,10,11]. The genes involved in embryo development regulation (such as Hox genes) are especially interesting for this purpose, because they are often retained after duplications and receive new functions. Besides, it was previously shown that fluorescence in situ hybridization (FISH) with chromosome-specific probes [12,13] and repetitive elements [8,14] also might be very useful for detecting extended paralogous regions in polyploids. Moreover, the chromosome-specific libraries used for probe generation can be sequenced and aligned to the reference genome [15], providing information on gene content and revealing syntenic blocks. This approach allowed revealing the regions of homology between B-chromosomes and the host genome and seems to be promising for polyploid species, although it has never been applied for such objects so far.

Representatives of the order Acipenseriformes are convenient objects to study the genome evolutionary transformations following WGDs, as this taxon has existed at least since the Early Jurassic [16]. Acipenseriformes are included in ray-finned fishes (Actinopterygii) and form a sister lineage to Holostei and Teleostei with a divergence time of about 360 Mya [17].

One of the features of this group is that all extant species are derived from a putative 60-chromosomal non-polyploid ancestor [18,19] after one or several independent ancestral WGDs [13,20]. The lowest chromosome number among modern acipenserids is about 110–120 chromosomes [18]; besides, additional rounds of polyploidization have occurred in several lineages within the family [13,18,19], resulting in diploid chromosome numbers of 360 or even 380 in some species [18,21]. An interesting, but yet understudied, problem is to reveal paralogous chromosomes derived by the duplication of ancestral protochromosomes and to track their evolutionary path after the polyploidization event [13].

In this study, we focused on sterlet (*Acipenser ruthenus*, ARUT), a small sturgeon with 2n = 120, recently characterized by an ongoing rediploidization process after the ancestral acipenserid specific WGD [12,18]. We obtained a set of sterlet chromosome-specific microdissection libraries and sequenced them on the Illumina MiSeq platform (Illumina, San Diego, CA, USA). After alignment to the reference genome of the spotted gar (*Lepisosteus oculatus*, LOC), we predicted syntenic blocks between the two species. It is important to note that the spotted gar (a ray-finned fish, the lineage of which is a sister to Teleostei) did not undergo any WGDs after 2R [10].

As the evolutionary distance between sturgeons and gars is very high (divergence up to 360 Mya [17]), we also performed an alignment of the sequenced canine chromosome-specific library to a set of reference genomes from dog to spotted gar (divergence over 450 Mya) to validate the power of conserved synteny prediction at various evolutionary distances.

## 2. Materials and Methods 

### 2.1. Samples Origin

All sterlet specimens used here were obtained from fish farms and described previously [12]. Microdissection-derived chromosome-specific libraries were obtained from the single sterlet female of Yenisei origin (Specimen 12 in [12]). FISH experiments were performed on metaphase chromosomes of two sterlet males from Irtysh river (specimens (Specimens 7 and 9) and two females from Irtysh and Yenisei (Specimens 6 and 12). Cot DNA for pre-hybridization was obtained from the single female of Irtysh origin (Specimen 6).

### 2.2. Chromosome Preparation, Staining, Probe Generation and Painting

**Figure 1:**
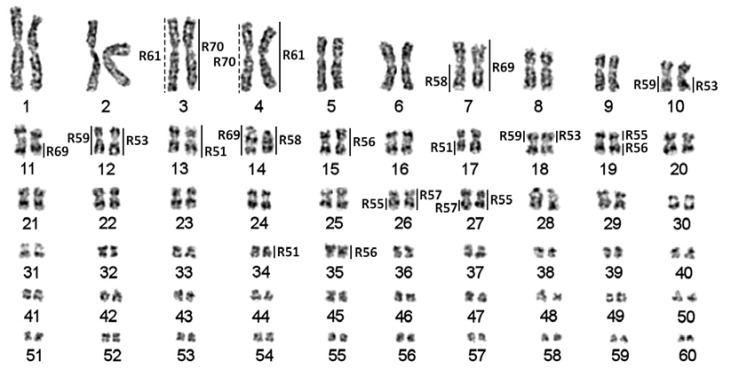
Sterlet karyotype [12] with assigned localization of chromosome-specific libraries. Dotted lines correspond to weak signals.

We used previously-described chromosome suspensions from the established cell lines [12]. Microdissection was performed according to [22]. Single macro- and mid-sized chromosomes were microdissected in this study (Figure 1). The chromosome libraries and FISH probes were generated as described before [12] using Sigma-Aldrich WGA kits (Sigma-Aldrich, Saint Louis, MO, USA). The prepared libraries were purified and stored at –20 °C. We used “R” in library names to not confuse them with the numbers of chromosomes.

GTG (G-banding by trypsin using Giemsa) staining, FISH and microscopy analysis were performed as previously described [12]. To identify the chromosome of origin, as well as paralogous regions, we carried out dual-color FISH with different probes in a series of pairwise experiments and compared the sizes, morphology and banding pattern of labeled chromosomes.

### 2.3. Next Generation Sequencing

Libraries were prepared according to the Illumina TruSeq protocol for the HT Sample Preparation Kit (Illumina) skipping the DNA fragmentation step. 300 bp paired-end reads were generated on Illumina MiSeq using the Illumina MiSeq Reagent Kit v3 according to the manufacturer’s instructions.

### 2.4. Alignment of the Sterlet Chromosome-Specific Library to the Spotted Gar Genome

The analysis was performed with the degenerate oligonucleotide-primed (DOP)seq analyzer pipeline [15]. Briefly, paired reads were trimmed to remove whole genome amplification (WGA) primers, aligned to the spotted gar genome (GCA_000242695.1) with Bowtie 2 (“--very-sensitive-local” profile) [23] or Burrous-Wheeler Aligner (BWA)-MEM (default options) [24], and after that, human contaminant reads and repetitively mapped reads (mapping quality (MAPQ) < 20) were removed.

Regions of gar linkage groups showing the smallest pairwise distances between mapped read positions were considered as the target (i.e., present on sterlet chromosomes). Regions shorter than 4 kbp and regions with less than 20 mapped positions were not considered.

### 2.5. Alignment of the Dog Chromosome-Specific Library to the Genomes of Vertebrate Species

We used published sequencing data for the flow sorting derived canine chromosome 12 library (*Canis lupus familiaris* chromosome 12 (CFA12), read archive SRA:PRJNA285957) [15] to evaluate how alignment quality depends on evolutionary divergence times. A total of 373,169 Illumina reads for CFA12 was analyzed, and a set of reference genomes was used: dog (GCA_000002285.2), cat (*Felis catus*, FCA, GCA_000181335.3), cattle (*Bos taurus*, BTA, GCA_000003055.4), human (*Homo sapiens*, HSA, GCA_000001405.25), gray short-tailed opossum (*Monodelphis domestica*, MDO, GCF_000002295.2), chicken (*Gallus gallus*, GGA, GCA_000002315.3), spotted gar and zebrafish (*Danio rerio*, DRE, GCA_000002035.3). The analysis was performed with the DOPseq analyzer pipeline [15] using DOP-PCR primer trimming, BWA-MEM alignment algorithm, minimum MAPQ = 20 and without contaminant read filtering.

## 3. Results

### 3.1. Characterization of Microdissection-Derived Libraries

Following the previously-proposed nomenclature [14], the 10 largest chromosomes in the sterlet karyotype were referred to as macrochromosomes; the chromosomes ARUT11–30 were referred to as mid-sized chromosomes, and ARUT31–60 (highly enriched with repetitive DNA) were referred to as microchromosomes (Figure 1). 

**Figure 2:**
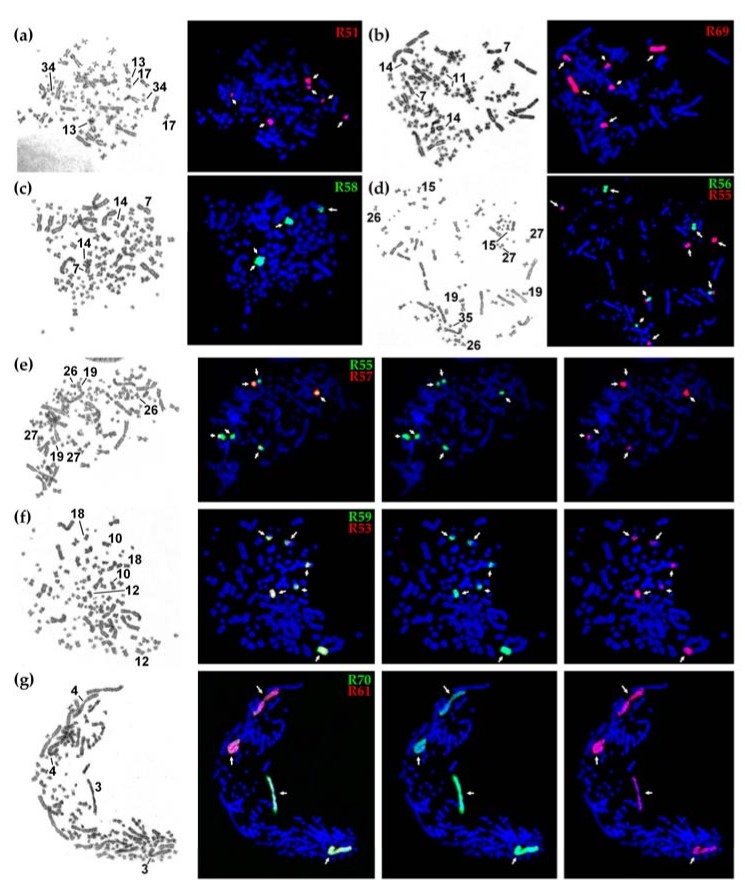
The results of fluorescence in situ hybridization (FISH) with sterlet chromosome-specific libraries: (**a**–**d**) G-banding by trypsin using Giemsa (left) and localization of FISH probes (right); (**e**–**g**) localization of the probes giving overlapping signals, from left to right: GTG-banding, dual-color FISH of two probes, localization of biotin-labeled probe (green), localization of digoxigenin-labeled probe (red).

From over 90 sterlet chromosome-specific libraries obtained using microdissection. Here, we focused on ten libraries derived from macro- and mid-sized chromosomes of the karyotype, leaving hardly identifiable smaller chromosomes for subsequent studies. As sterlet has a high number of chromosomes (2n = 120), which are difficult to distinguish due to weak banding and similar morphologies (most of the sterlet chromosomes are metacentric), we microdissected anonymous chromosomes and identified the position in the karyotype only after subsequent FISH experiments (Figure 1 and Figure 2). The libraries analyzed here can be divided into two groups: macrochromosomal probes R61 and R70 revealed four chromosomes each marking two pairs of paralogous chromosomes (Figure 2g) (the same pattern was previously observed for other macrochromosomes [12]); the probes of mid-sized chromosomes (R51, R53, R55, R56, R57, R58, R59) and the ARUT7 (R69) probe mostly produced more than four signals (Figure 2a–f), suggesting some interchromosomal rearrangements.

FISH experiments with chromosome-specific probes disclosed some features of sterlet paralogous chromosomes. In the absence of any interchromosomal rearrangements after the tetraploidization event, it is expected that one probe hybridizes with two (in the case when one paralogous copy is degenerated) or four chromosomes (two pairs of paralogs) [12]. Here, we can see that probes R61 and R70 both hybridize with whole ARUT3 and ARUT4, with R61 labelling ARUT4 and R70 labelling ARUT3 more intensively, suggesting that R61 and R70 were obtained from different paralogs, ARUT4 and ARUT3, respectively (Figure 2g).

However, the hybridization pattern was more complicated for other libraries. Most of them hybridized with a whole mid-sized chromosome pair and additionally labelled other regions: either arms of one or two other mid-sized pairs or a whole microchromosome pair. This means that several interchromosomal rearrangements took place during over 100 million years of evolution after the ancestral sturgeon WGD event (acipenserid-specific WGD, ASGD) [13].

Thus, in the case of R51, the chromosome ARUT13 was microdissected resulting in painting of thewhole ARUT13 and its paralogous regions: ARUT17q and the whole ARUT34 marked (Figure 2a). Similarly, R55 labeled its original chromosome, ARUT27, and its paralogous regions, ARUT19p and ARUT26q (Figure 2d, red, and Figure 2e, green). R56 was derived from the ARUT15, and this chromosome or its paralog might have undergone a fission followed by subsequent fusion with one of the ARUT27 paralogous regions. R57 was obtained from ARUT26, and ARUT27q represents its paralog (Figure 2e, red). However, this paralogous region is significantly shorter than ARUT26. The remainder might be lost or too diverged. Libraries R53 and R59 were derived from the same chromosome pair (ARUT12), because their fluorescent probes produced identical staining patterns (Figure 2f). R58 was obtained from ARUT14, the largest acrocentric chromosome in the sterlet karyotype, with a paralogous region at ARUT7q (Figure 2b). The whole chromosome ARUT7 was also microdissected (library R69), and the resulting probe labelled ARUT7, ARUT14 and ARUT11q (Figure 2c).

### 3.2. Sequencing and Alignment

All 10 sampled libraries were sequenced on Illumina MiSeq, an additional round of sequencing with a higher number of reads was performed for the libraries R53, R58, R61, R69 and R70 (Table 1). Only about 0.77–2.16% of reads were aligned to the spotted gar genome using Bowtie 2. We assumed that high levels of sequence divergence between sterlet and gar genomes (360 Mya) might be responsible for low mapping rate. We thus utilized BWA-MEM, an algorithm designed to work with long reads (PacBio, Oxford Nanopore) with high sequencing error frequencies which also result in reduced homology to the reference. As a result, an acceptable 40.34–52.47% alignment rate was achieved. Detection of regions present on chromosomes was performed with a recently-proposed approach for classification of the reference genome based on the distances between mapped read positions [15]. This method was specifically designed to work with inherently incomplete sequencing data from single of input chromosomes and a biased amplification. The obtained results allowed for identification of relatively large syntenic regions and even breakpoints within gar chromosomes (e.g., for R51 and R57), independently of alignment algorithms or sequencing depths (Table 2). In the cases of libraries R61, R69 and R70 (larger chromosomes), the top target regions varied, and their pairwise distances were not much lower than the distances of other regions, suggesting a blurring of the homology signal due to higher sequence divergence, extensive rearrangements or enrichment with repetitive DNA (Table S1, Figure S1). Sequence data are available in NCBI SRA under Accession Numbers SAMN07665612-21.

To investigate how the evolutionary distance between the sampled species and the reference genome affects the efficiency of the algorithm for chromosomal region detection, we utilized the previously-sequenced library of the dog chromosome 12 [15]. Syntenic relations between dog and other mammalian species are characterized cytogenetically [25], and whole-genome alignment data are available for dog against cat, cow and human genomes [26]. For other species, orthology data were available for the human genome (Table 3).

Overall, chromosome region predictions remain in a good agreement with cytogenetic and comparative genomics data when alignments are performed within mammals. Alignment to more distant species results in lower power predictions. Alongside, mean alignment lengths are reduced, indicating to the probability of accidental mappings without the underlying true homology. Higher alignment lengths from true sequence matches were also observed in target chromosomes for sterlet vs. gar data (Table S1).

However, alignment to the spotted gar genome still produces meaningful results: three regions of the five predicted seemingly represent true homology, and only two regions are missing. Lack of a specific signal upon the alignment to zebrafish genome is not surprising, given a teleost-specific WGD saturating the signals between two paralogous sequences and extensive rearrangements in teleosts resulting in decreased syntenic region sizes.

## 4. Discussion

This study presents the first comparison of Acipenseriformes (sterlet) and Lepisosteiformes (spotted gar) genomes on the chromosomal level. These two groups occupy basal positions among Actinopterygii, have low rates of evolution [10,30] and retain some ancestral traits [31], thus being substantial for evolutionary studies in vertebrates. It is also known that the spotted gar genome shares large syntenic chromosome segments even with tetrapods, with most of its linkage groups being similar to ancestral ones [10]. The spotted gar karyotype consists of 58 chromosomes; therefore, it also might be quite similar to the non-polyploid ancestor of Acipenseriformes (diploid number of about 60) [13]. As no genome assemblies or maps are yet available for Acipenseriformes, synteny analysis based on whole-genome alignment or gene order comparison remains impossible.

Our study allowed reconstructing syntenic relations between several sterlet chromosomes and linkage groups of the spotted gar genome. We focused on the sterlet chromosomes of large (ARUT3, 4 and 7) and medium (ARUT12, 14, 15, 18, 26 and 27) sizes. Recovery of similar spotted gar genome regions from independent libraries derived from ARUT12 (R53, R59), as well as reasonable results of dog-to-gar analysis justified the applicability of the chosen method for chromosome comparisons between species diverging up to 450 Mya thanks to synteny conservation in most vertebrate lineages.

**Figure 3:**
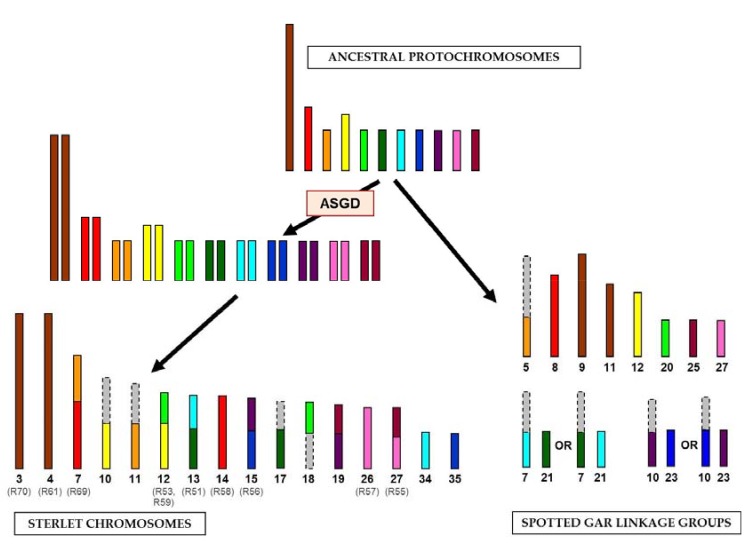
The scheme of evolutionary chromosomal rearrangements between sterlet and spotted gar. The colored bars correspond to the regions of synteny, revealed in this study; the gray bars correspond to the regions with unrevealed synteny. The alternative evolution pathways are shown for spotted gar linkage groups 7, 10, 21 and 23. The names of chromosome-specific libraries are given in parentheses. The sizes of chromosomes and linkage groups are shown roughly. ASGD, acipenserid-specific genome duplication.

The identification of large syntenic regions between genomes of basal Actinopterygii lineages confirmed the hypothesis of relative karyotype conservation in sturgeons [13]. All samples, except for ARUT14 and ARUT23, revealed homologous regions on two or more spotted gar linkage groups, suggesting that chromosomal fusions and fissions occurred in sterlet and spotted gar since these species diverged 360 Mya. These rearrangements could be attributed to either the gar or sturgeon lineage by comparison of the sterlet reads to spotted gar genome alignments, synteny data for spotted gar and chicken (a representative tetrapod species with very conserved linkage groups) [10] and a reconstructed bony vertebrate (Osteichthyes) ancestor chromosomes [19]. The revealed hypothetical chromosomal rearrangements are shown in Figure 3. The sizes of ancestral chromosomes, sterlet chromosomes and spotted gar linkage groups can only be roughly estimated, but most of the depicted spotted gar genome regions (LOC20, LOC21, LOC23, LOC25, LOC27 and distal parts of LOC5, LOC7 and LOC10) actually have similar sizes of about 14–17 Mbp (Table S1, [10]).

Pairs of paralogous sterlet chromosomes demonstrate similar homology signals in the spotted gar genome. ARUT3 and ARUT4 are paralogous according to FISH results and share two homologous spotted gar regions (LOC9 and LOC11), but also have differing ones (Table 2), which may be an artifact of the method or true differences not revealed by FISH. It should be noted that two shared target regions correspond to the same chicken chromosome (GGA2), which most likely represents the ancestral state [19]. Therefore, we assume that the ancestral protochromosome underwent a fission in spotted gar and was duplicated in sterlet (Figure 3). With regard to the libraries labelling a whole chromosome pair and some additional regions (R51, R53, R55, R56, R57, R58, R59 and R69), it is evident that at least one of the paralogous chromosomes is rearranged, and comparison to the ancestral chromosomes will reveal which of the paralogs retained an ancestral state (Figure 3). 

Despite the fact that acipenserids with a similar ploidy level possess similar diploid numbers, some authors assumed species-specific chromosomal rearrangements based solely on the number and distribution of nucleolus organizer regions (NORs) [32]. Ludwig et al. also suggested that species-specific chromosomal rearrangements took place in acipenserid evolution, as those might have prevented paralogous chromosome synapsis in meiosis, thus decreasing quadruplex formation and proper segregation failure [18]. Inversions (often undetected using FISH with whole chromosome probes or alignment of chromosome-specific probes on different species) might also have played an important role in sturgeon karyotype evolution, as was shown in many teleost species [7,33,34], amphibians [8,35] and mammals [36,37].

A recent genomic project on allopolyploid *Xenopus laevis* has demonstrated that two subgenomes evolved differently after the WGD. While one of the parent genomes remains conserved, the chromosome of the second subgenome evolved much faster and underwent multiple rearrangements and pseudogenization [8]. Although we still do not know whether auto- or allo- polyploidization took place in the sturgeon ancestor, an uneven accumulation of tandemly-arranged repetitive elements took place on paralogous chromosomes [14]. It is noteworthy that chromosomes of different sizes underwent specific evolutionary pathways: the largest karyotype elements seem to be much more conserved in sterlet evolution than the smaller elements, and a similar process has been described in birds [38] and reptiles [39,40]. Accumulation of repetitive elements was also different in various sterlet chromosome groups with large chromosomes accumulating interstitial repetitive blocks and small chromosomes enriched in dispersed repeats and accumulating pericentromeric tandem repetitive sequences [14].

## 5. Conclusions

To sum up, the sterlet karyotype, although it consists of 120 chromosomes, does not represent just a duplicated karyotype of the acipenseriform ancestor. A high number of interchromosomal rearrangements took place after ASGD. However, the fact that acipenserids easily hybridize and produce fertile offspring implies that most rearrangements have probably occurred in the acipenserid common ancestor. Additional molecular cytogenetic and genomic studies are necessary to investigate the complex genome evolution after WGD events in different acipenseriform species. The method of isolated chromosome sequencing coupled with FISH on other high ploidy level sturgeon species, as well as whole-genome-level synteny analysis would help characterize the karyotype evolution in sturgeons in a more detailed way.

## Figures and Tables

**Table 1:** Statistics of sequencing and mapping of sterlet chromosome-specific libraries. Total reads: number of reads in all sequence rounds; # reads: initial number of reads in a sequence round; # aligned reads: number of reads significantly mapped (mapping quality (MAPQ) ≥ 20)) to spotted gar genome with the given alignment algorithm, Bowtie 2 or Burrous-Wheeler Aligner (BWA)-MEM.

Library	Total Reads	First Round of Sequencing			Second Round of Sequencing			Combined Alignment
		# Reads	# Aligned Reads, Bowtie 2	# Aligned Reads, BWA-MEM	# Reads	# Aligned Reads, Bowtie 2	# Aligned Reads, BWA-MEM	# Aligned Reads, BWA-MEM
R51	136,912	136,912	1806	66,894	-	-	-	-
R53	291,544	85,926	1015	43,365	205,618	1582	92,913	136,288
R55	100,975	100,975	1077	47,303	-	-	-	-
R56	109,161	109,161	1064	57,278	-	-	-	-
R57	66,669	66,669	968	30,541	-	-	-	-
R58	369,795	83,882	1493	37,870	285,913	4102	118,337	156,226
R59	98,724	98,724	1493	44,889	-	-	-	-
R61	208,810	78,743	1702	35,949	130,067	2364	57,796	93,743
R69	225,362	64,839	1048	29,142	160,523	1980	66,237	95,380
R70	213,732	42,616	826	19,336	171,116	2456	69,026	88,356

**Table 2:** Characterization of sterlet chromosome-specific libraries. Chromosome of origin and paralogous segments are revealed by FISH. Homology to spotted gar linkage groups revealed based on library sequencing. ARUT, *Acipenser ruthenus*; LOC, *Lepisosteus oculatus*.

Libraries	ARUT Microdissected Chromosome	FISH Signals	Top Target Regions of LOC	Chicken Chromosomes [10]	Ancestral Amniote Chromosomes [19]	Ancestral Bony Vertebrate Chromosomes [19]
R51	ARUT13	ARUT13, ARUT17q, ARUT34	LOC7 (distal region)	5	7 or 13 or 14	8 or 15 or 16
			LOC21	17	3 (part) or 26	3
R53, R59	ARUT12	ARUT10q, ARUT12, ARUT18p	LOC12	7	4 (part)	2 (part)
			LOC20	15	11	12
R55	ARUT27	ARUT19p, ARUT26q, ARUT27	LOC25	21	22	25
			LOC27	5	7 or 13 or 14	8 or 15 or 16
R56	ARUT15	ARUT15, ARUT19q, ARUT35	LOC10 (distal region)	18	1 (part)	6 (part)
			LOC23	11	12	14
R57	ARUT26	ARUT26, ARUT27q	LOC27	5	7 or 13 or 14	8 or 15 or 16
R58	ARUT14	ARUT7q, ARUT14	LOC8	1	1 or 4	2 (part) or 5 or 23
R61	ARUT4	ARUT3 (weak), ARUT4	LOC9	2	2	1
			LOC11			
			LOC16	3	5 or 1 or 13 or 14	4 or 15 or 16
R69	ARUT7	ARUT7, ARUT14, ARUT10q	LOC5 (distal region)	6	6	17
			LOC8	1	1 or 4	2 (part) or 5 or 23
R70	ARUT3	ARUT3, ARUT4 (weak)	LOC9	2	2	1
			LOC11			
			LOC19	28	20	22
			LOC25	21	22	25

**Table 3:** Alignment of chromosome-specific dog library CFA12 to various reference genomes. CFA, *Canis lupus familiaris*; FCA, *Felis catus*; BTA, *Bos taurus*; HSA, *Homo sapiens*; MDO, *Monodelphis domestica*; GGA, *Gallus gallus*; LOC, *L. oculatus*; DRE, *Danio rerio*. References are given for synteny information and divergence estimates.

Alignment Results	Reference Genomes							
	CFA	FCA	BTA	HSA	MDO	GGA	LOC	DRE
Aligned reads	330,701	213,039	172,411	180,466	137,621	193,115	208,142	216,574
Collapsed positions	36,073	34,781	25,688	32,951	17,452	24,822	26,229	29,875
Recovery, bp	2,743,657	2,178,208	919,150	1,909,100	424,206	519,302	537,072	620,913
Reads per position	9.2	6.1	6.7	5.5	7.9	7.8	7.9	7.2
Position mean size, bp	76	63	36	58	24	21	20	21
Identified regions	12	B2	9, 23	6	2	27, 5, 3, 2	16, 7, 8, 1, 11	4
Syntenic regions for CFA12	12	B2 [25,26]	9, 23 [26]	6 [25]	2 [26]	3, 26 [26]	1, 16, 9, 11 [10]	13, 16, 19, 20, 21, 23 [27,28]
Approximate divergence time, Mya	0	55 [29]	85 [29]	90 [29]	180 [19]	350 [4]	450 [4]	450 [4]

## Supplementary Materials

The following are available online at www.mdpi.com/2073-4425/8/11/318/s1. Table S1: The alignment results of sequenced sterlet chromosome-specific libraries; Figure S1: Distances between mapped read positions (X, coordinate in spotted gar linkage groups; Y, long scale distances; black dots, individual observations; red lines, mean values estimated by the region_dnacopy script).
