# Supplementary figures and images for: Next Generation Sequencing of Chromosome-Specific Libraries Sheds Light on Genome Evolution in Paleotetraploid Sterlet (Acipenser ruthenus)
Source: Genes (Basel). 2017 Nov 10;8(11):318. doi: 10.3390/genes8110318 (PMC5704231; doi:10.3390/genes8110318)

# R51.LepOcu1

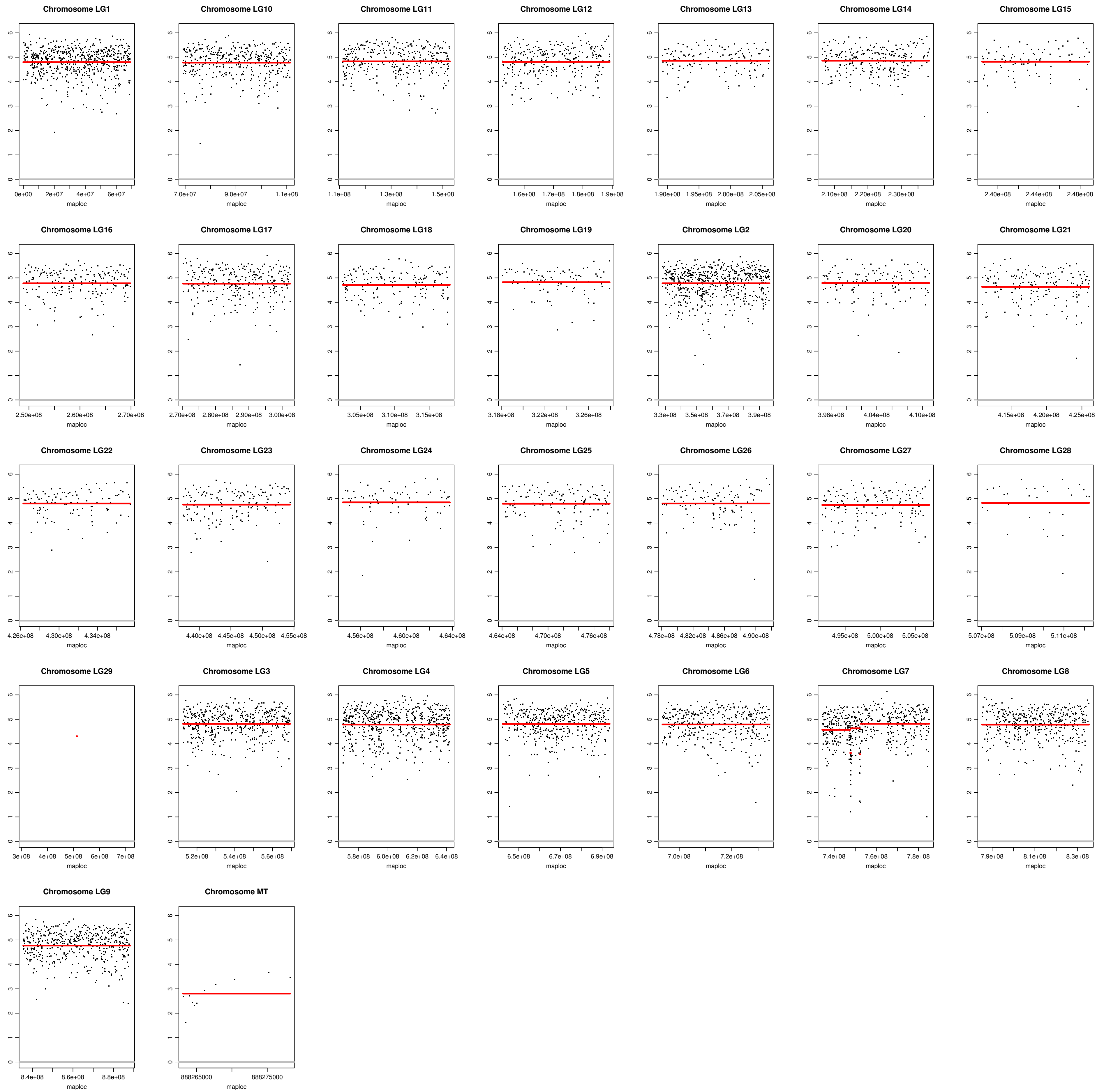

# R53.LepOcu1

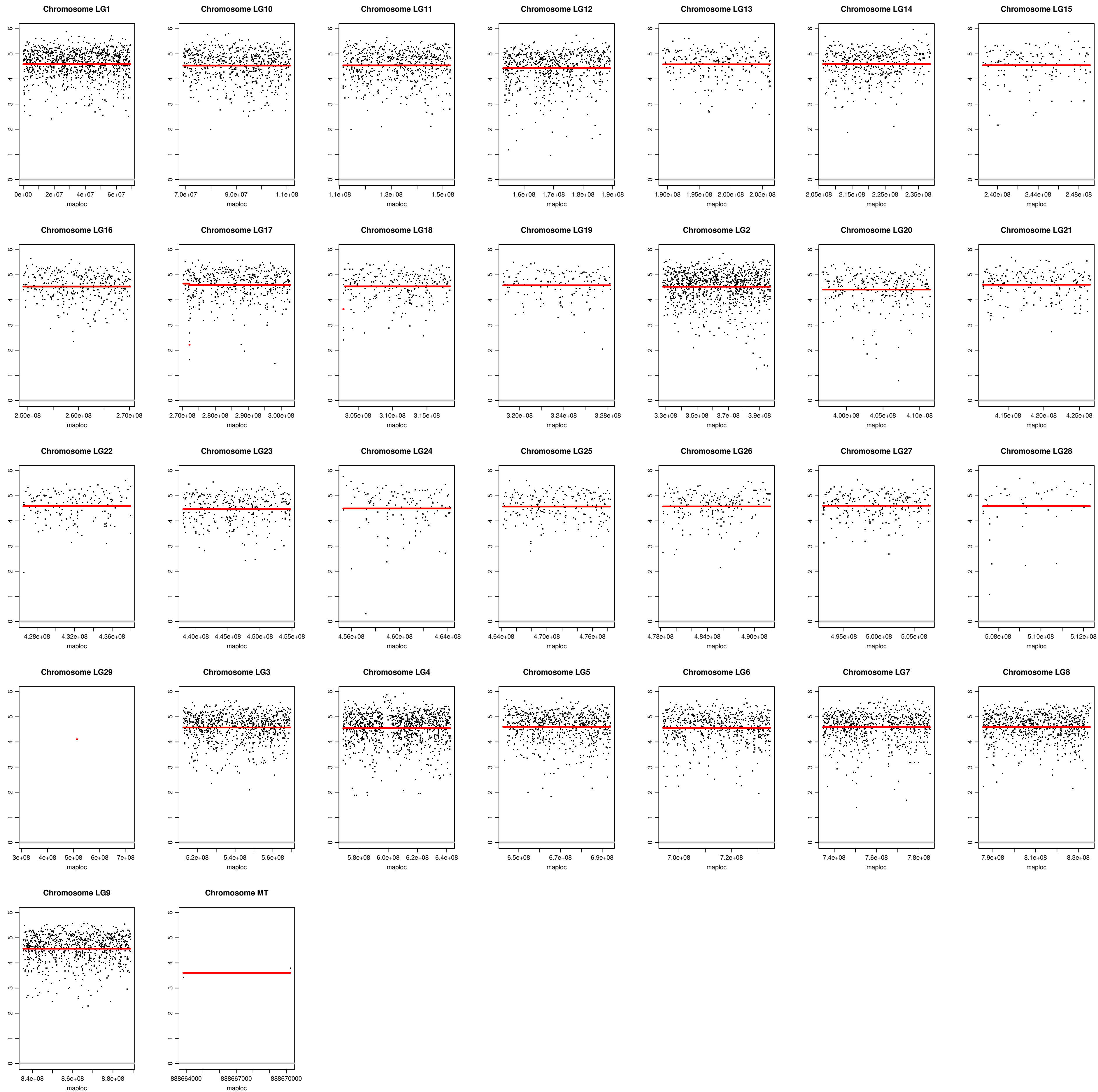

# R55.LepOcu1

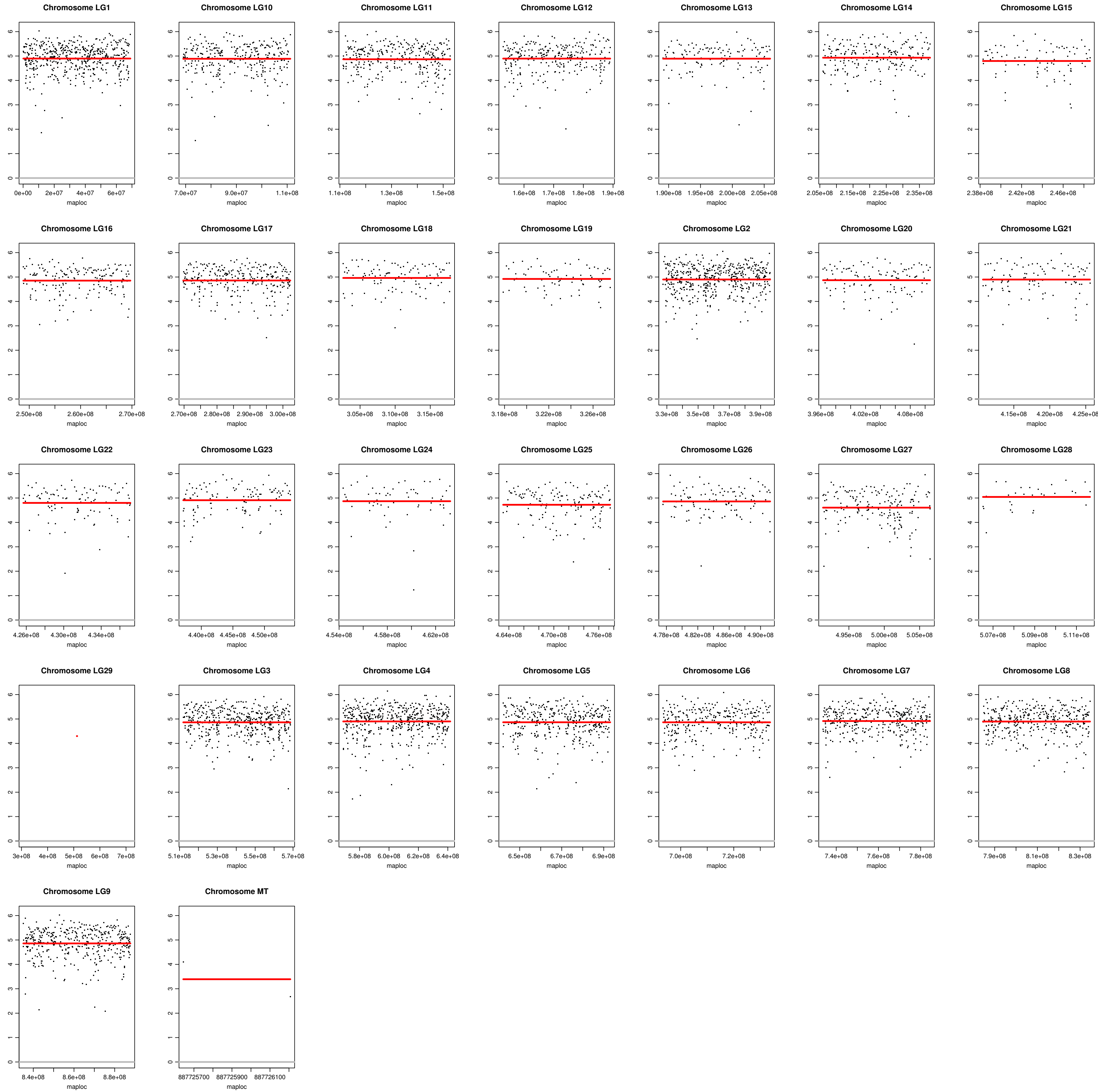

R56.LepOcu1

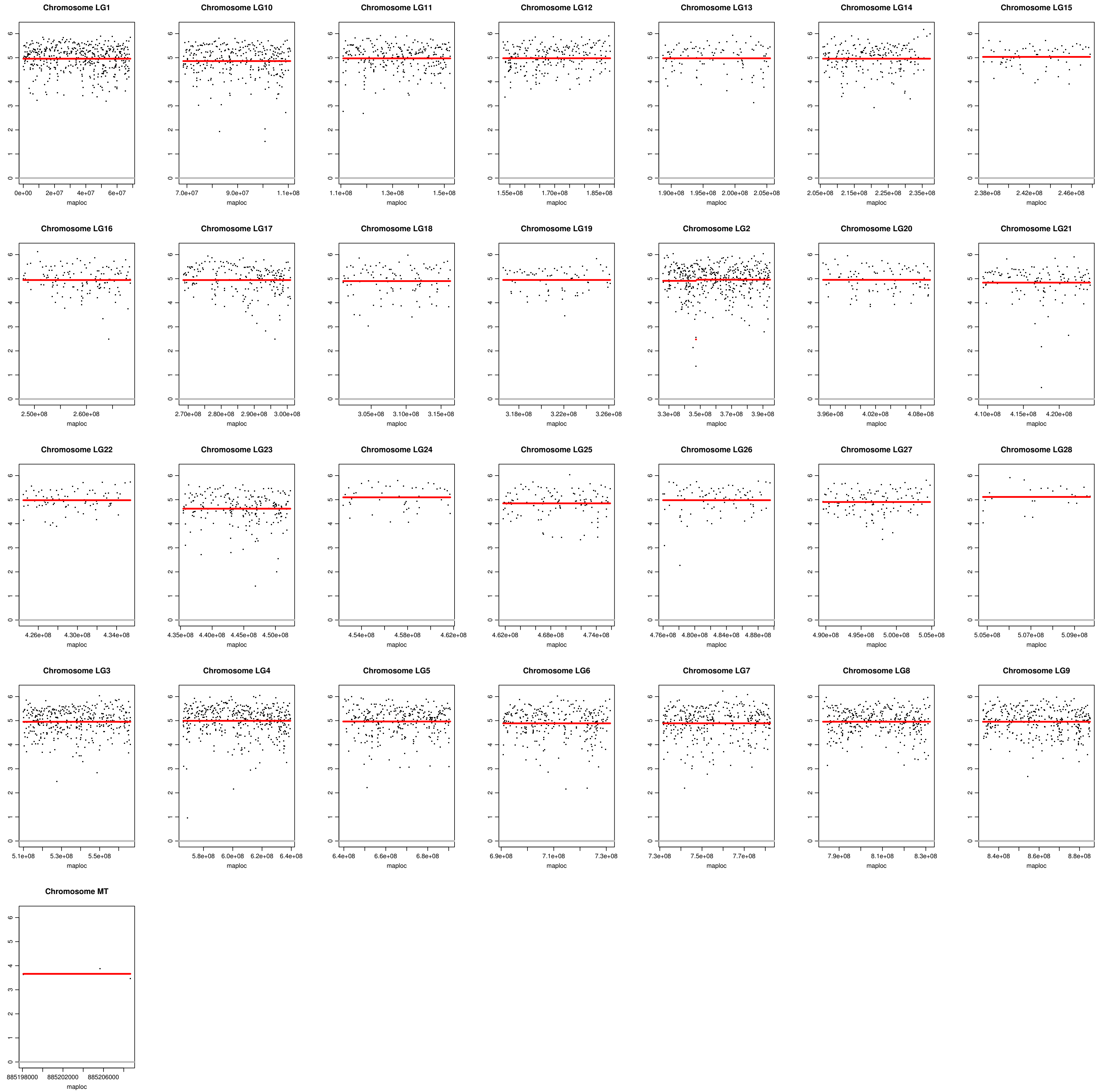

# R57.LepOcu1

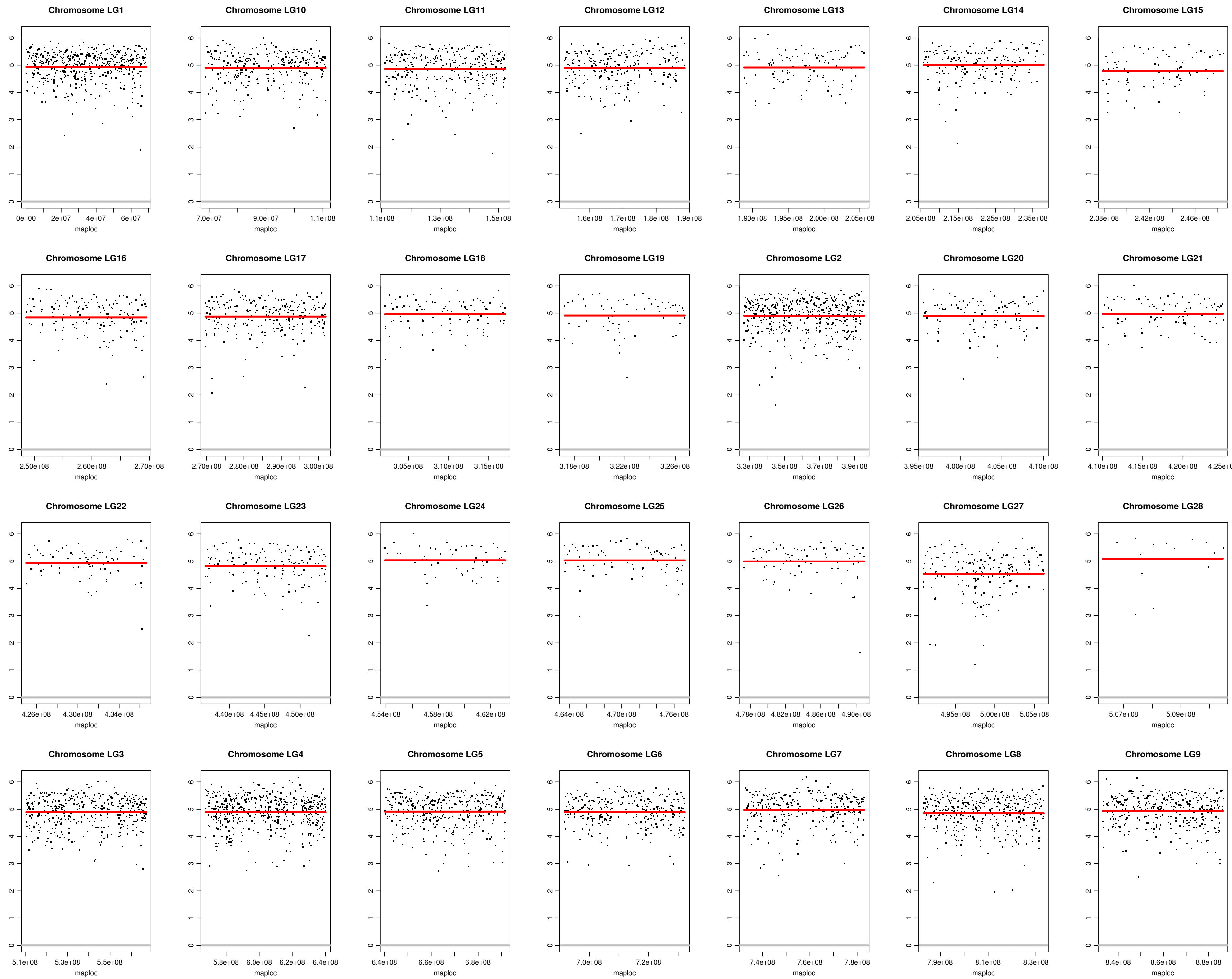

# R58.LepOcu1

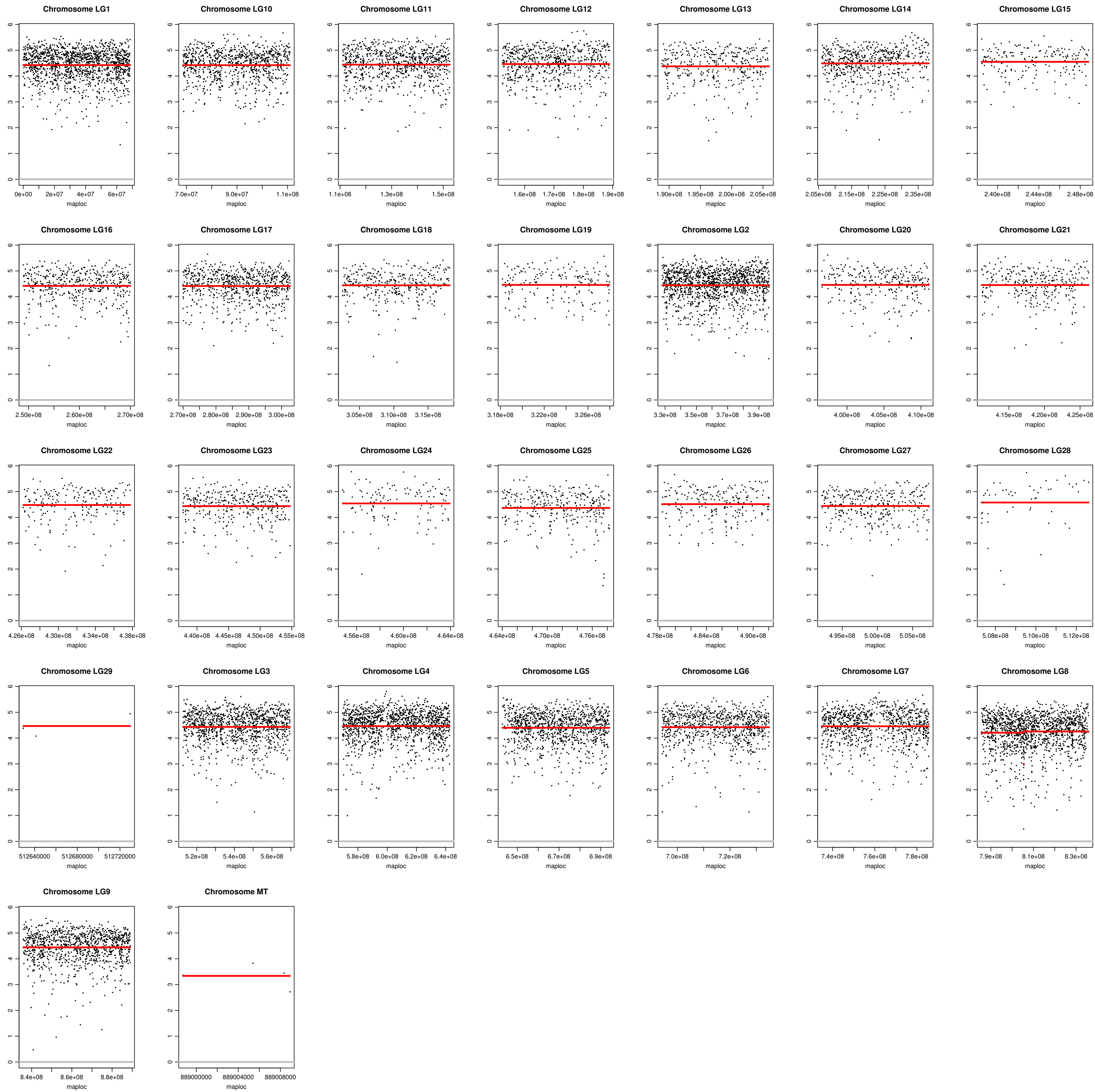

# R59.LepOcu1

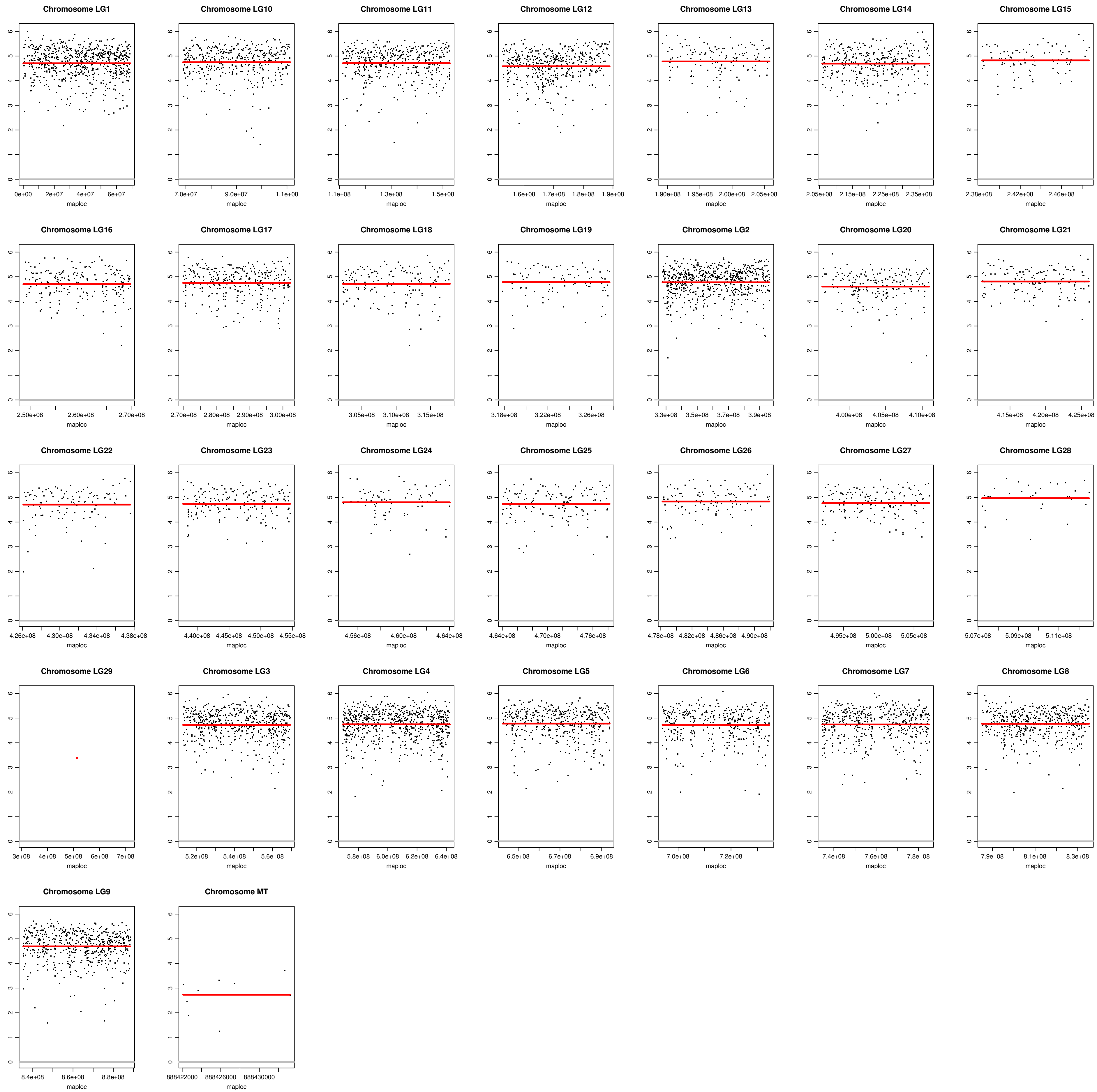

# R61.LepOcu1

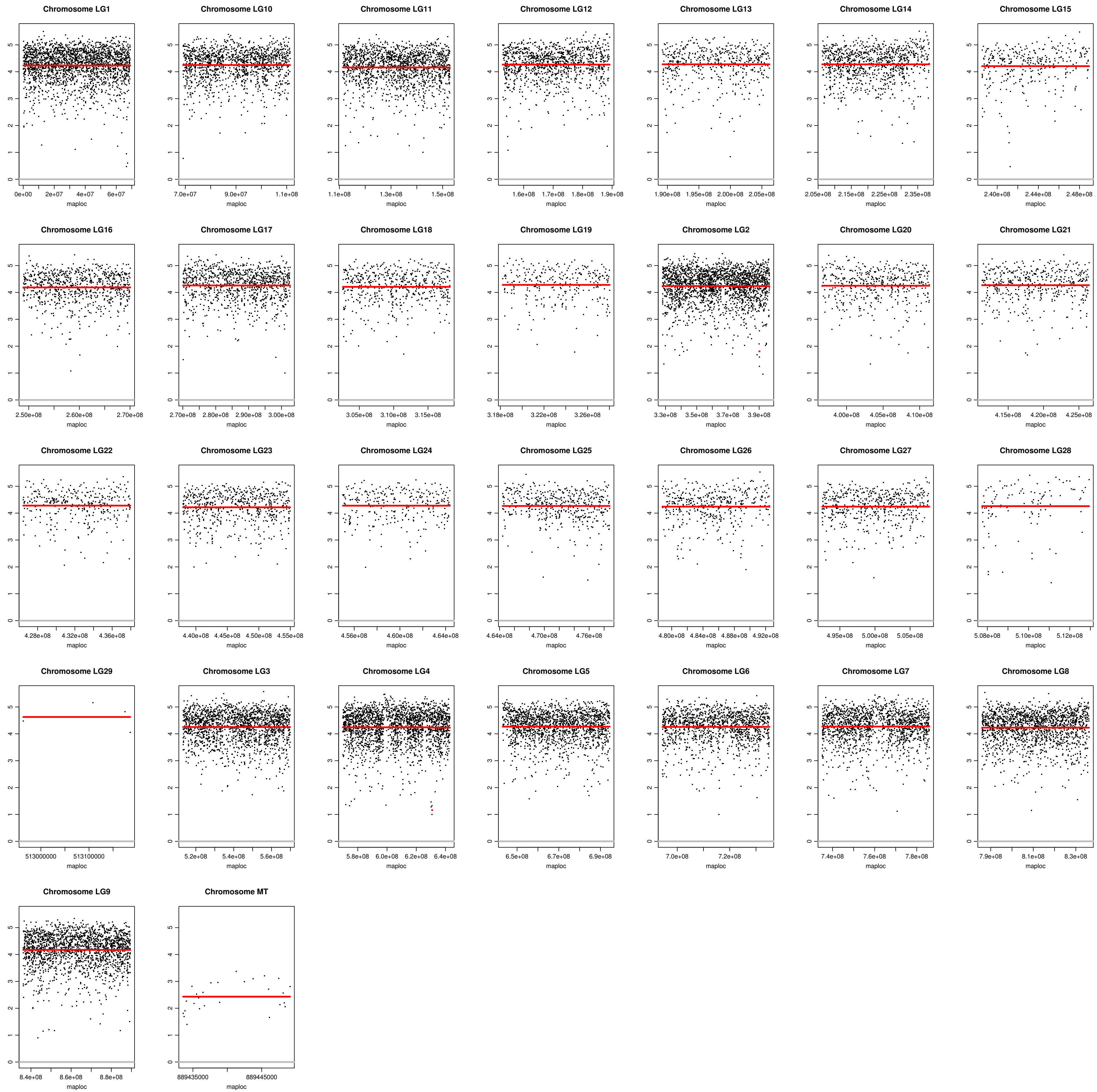

R69.LepOcu1

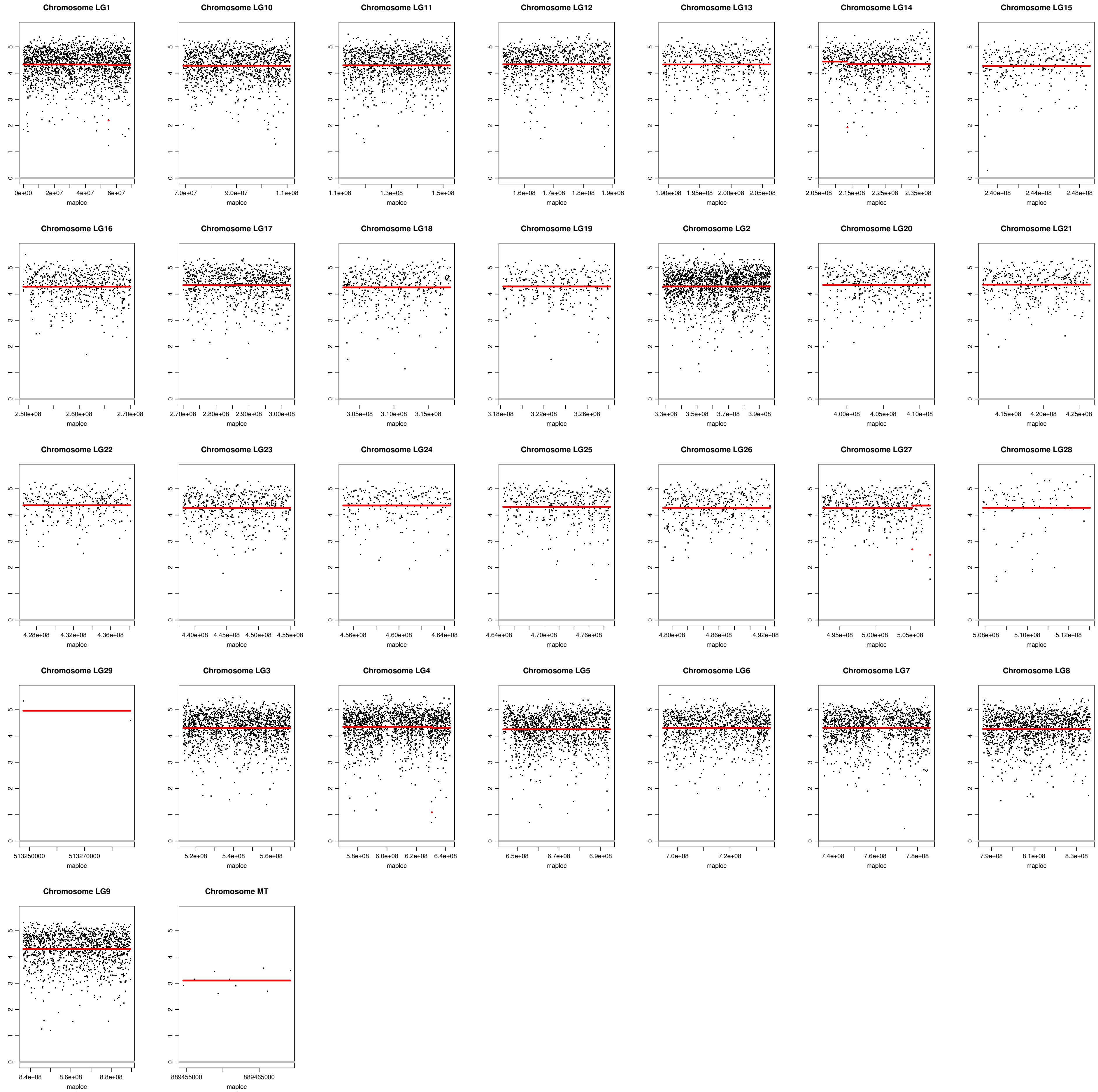

R70.LepOcu1

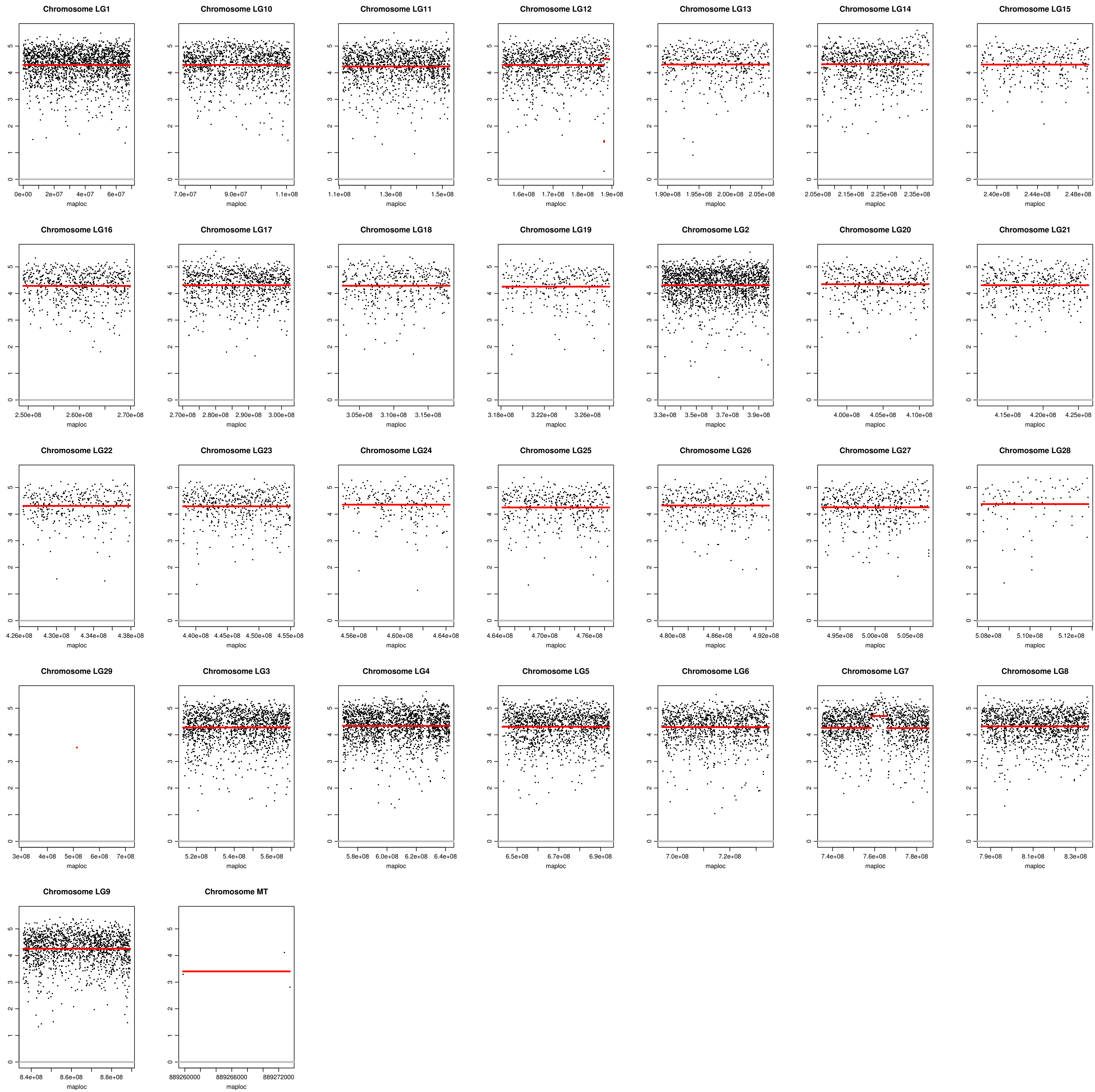

Supplement: Supplementary file 1 [file genes-08-00318-s001.zip › Supplementary/Document S2.pdf]
